# Case Report: Activated PI3-kinase-δ syndrome and ovarian malignancies: a case series from the European ESID-APDS registry

**DOI:** 10.3389/fimmu.2025.1572194

**Published:** 2025-04-30

**Authors:** Maria Pia Esposto, Nizar Mahlaoui, Hassan Abolhassani, Koen Van Aerde, Simone Cesaro, Anita Chandra, Stephan Ehl, Sven Kracker, Felipe Suarez, Vincent Barlogis, Alice Parisi, Maria Elena Maccari, Matteo Chinello

**Affiliations:** ^1^ Pediatric Hematology Oncology, Department of Mother and Child, Azienda Ospedaliera Universitaria Integrata Verona, Verona, Italy; ^2^ Pediatric Immuno-Haematology and Rheumatology Unit, Necker Enfants Malades University Hospital, Assistance Publique-Hôpitaux de Paris (AP-HP), Paris, France; ^3^ French National Reference Center for Primary Immune Deficiencies (CEREDIH), Necker Enfants Malades University Hospital, Assistance Publique-Hôpitaux de Paris (AP-HP), Paris, France; ^4^ Division of Immunology, Department of Medical Biochemistry and Biophysics, Karolinska Institutet, Stockholm, Sweden; ^5^ Research Center for Immunodeficiencies, Pediatrics Center of Excellence, Children’s Medical Center, Tehran University of Medical Sciences, Tehran, Iran; ^6^ Department of pediatric infectious disease and immunology, Amalia Children’s Hospital, Radboudumc, Nijmegen, Netherlands; ^7^ Department of Clinical Immunology, Cambridge University Hospitals NHS Foundation Trust, Cambridge, United Kingdom; ^8^ Department of Medicine, University of Cambridge, Cambridge, United Kingdom; ^9^ Institute for Immunodeficiency, Center for Chronic Immunodeficiency, Medical Center-University of Freiburg, Faculty of Medicine, University of Freiburg, Freiburg, Germany; ^10^ Laboratory of Lymphocyte Activation and Susceptibility to Epstein Barr Virus (EBV) infection, Imagine Institute, INSERM UMR 1163, Université Paris Cité, Paris, France; ^11^ Université Paris Cité, Inserm U-1163, Institut Imagine, Laboratoire of Hematological Disorders, Paris, France; ^12^ Service d’Hématologie Adulte and Centre de référence des déficits immunitaires héréditaires (CEREDIH), AP-HP, Hôpital Necker-Enfants Malades, Paris, France; ^13^ Department of Pediatric Hematology, Immunology and Oncology, APHM, Hôpital de la Timone Enfants, Marseille, France; ^14^ CEReSS Research Unit EA 3279 and Department of Public Health, Aix Marseille University, School of Medicine, Marseille, France; ^15^ Aix Marseille University, School of Medicine, Marseille, France; ^16^ Department of Pathological Anatomy, Azienda Ospedaliera Universitaria Integrata Verona, Verona, Italy; ^17^ Department of Pediatric Hematology, Oncology and Stem Cell Transplantation, Children’s Hospital, Medical Center – University of Freiburg, Faculty of Medicine, University of Freiburg, Freiburg, Germany

**Keywords:** activated PI3-kinase-δ syndrome, ovarian cancer, ovarian malignancies, IEI, inborn errors of immunity, female, cancer predisposition

## Abstract

Activated phosphoinositide-3-kinase-delta (PI3Kδ) syndrome (APDS) is an autosomal dominant inborn error of immunity (IEI) characterized by combined immunodeficiency and immune dysregulation with increased risk for lymphoma and other non-lymphoid malignancies. We describe five patients with ovarian malignancies among 110 female APDS patients participating in the European Society for Immunodeficiencies (ESID) registry and identified three additional cases in the literature. These findings document a relevant predisposition to these non-hematological malignancies in APDS patients.

## Introduction

Activated phosphoinositide-3-kinase-delta (PI3Kδ) syndrome (APDS) is an autosomal dominant inborn error of immunity (IEI) first described in 2013 (1, 2). PI3Kδ is part of the PI3K/AKT/mTOR/S6K signaling pathway implicated in cell survival, proliferation, and differentiation (3). APDS is caused by germline heterozygous gain of function (GOF) mutations in *PIK3CD* that encode for the catalytic subunit p110δ (APDS1) or loss of function mutations in *PIK3R1* encoding for the regulatory subunit p85α (APDS2) (4, 5). Mutations in the known tumor suppressor gene *PTEN*, a negative regulator of this signaling pathway, lead not only to a cancer predisposition syndrome but also to various degrees of APDS-like immune alterations (6–8). Increased PI3Kδ activity results in combined immunodeficiency and immune dysregulation. APDS is characterized by childhood onset with frequent sinopulmonary infections, severe or persistent herpes-family viruses’ infection, benign lymphoproliferation, autoimmunity, and increased risk for lymphoma and other malignancies (9). Immunological features are variable, class G immunoglobulins (IgG) and class A immunoglobulins (IgA) can be normal or low, while class M immunoglobulins (IgM) can be normal or high with a hyper-IgM phenotype. Vaccine responses are often impaired. Lymphopenia occurs in approximately one-third of patients, low B-cell or elevated transitional B cells are frequent, and T-cell profiles often show a reduction in CD4+ cells with an inverted CD4/CD8 ratio. Mild natural killer (NK) cell deficiency may also be present (10). The most common malignancies reported in APDS are lymphomas; however, also non-hematological malignancies have been described, including ovarian neoplasms. Only three previous cases of ovarian dysgerminoma in patients with APDS have been reported in the literature to our knowledge (11–13). We describe a case series of APDS patients with ovarian malignancies from the European Society for Immunodeficiencies (ESID) registry.

## Case 1

**Figure 1:**
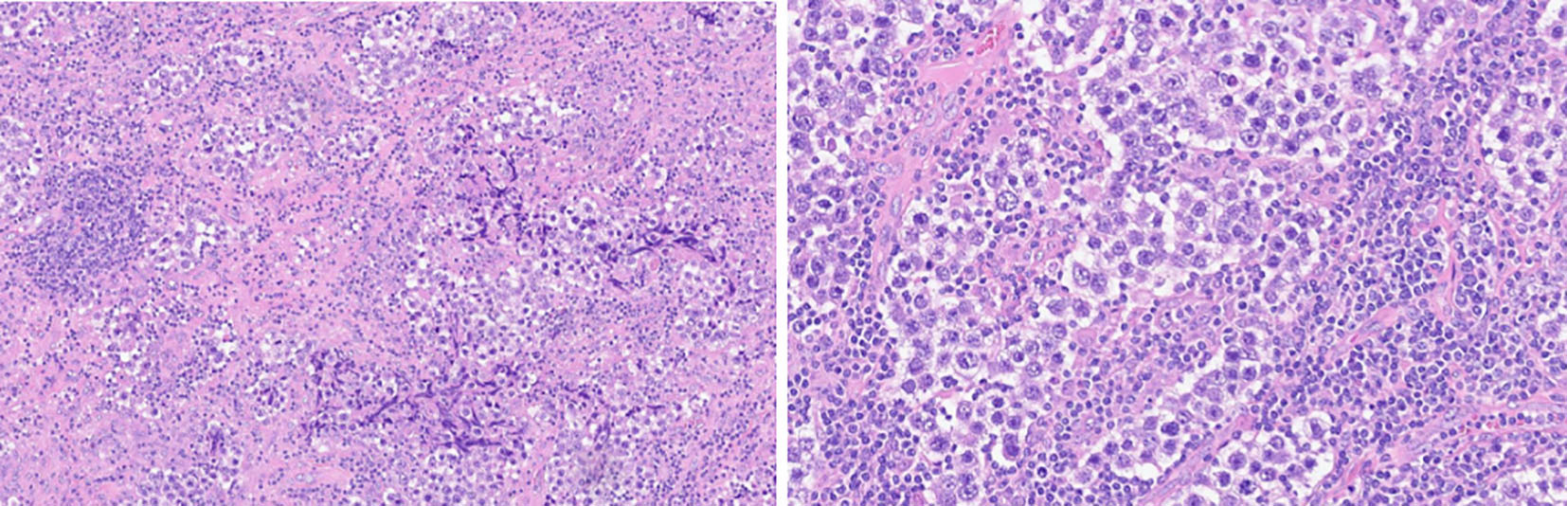
Ppatient 1: Biopsy histological features. hematoxylin eosin ×10 and ×20 magnification. Uniform rounded primitive germ cells with clear cytoplasm and macronucleoli, arranged in nests separated by thin fibrous septa containing lymphocytes. (immunophenotype: SALL4 pos, D2–40 pos, N1NK pos, EMA neg, 1G12 neg, HCG neg, CD30 neg, and CD117 cKit pos).

The proband is a 15-year-old female adolescent born in Italy to healthy non-consanguineous parents coming from Morocco after an uneventful pregnancy. No family history of IEI was reported. At the age of 9, she was admitted for significant gastrointestinal symptoms (vomiting, diarrhea, and abdominal pain without fever), and the abdominal ultrasound (US) and computed tomography scan (CT) showed extensive abdominal lymphadenopathy and moderate splenomegaly. She underwent a laparoscopic biopsy, and the histological study identified follicular lymphoid hyperplasia. The lymphocyte subset testing in peripheral blood revealed an increased percentage of alpha beta double-negative T cells (CD3+, TCRαβ+, CD4−, CD8−: 9.4%), so the patient was initially diagnosed with an autoimmune lymphoproliferative syndrome-like disorder (ALPS-like). She started immunosuppression with sirolimus as a therapeutic strategy with a good response. Some months later, the results of the genetic panel revealed one *de novo* heterozygous pathogenic variant in *PIK3CD* (c.3061G>A), so the diagnosis APDS1 was made (14). The therapeutic strategy remained the same. At the age of 13, during US follow-up, a right ovarian lesion was found. The magnetic resonance imaging (MRI) confirmed the lesion with a solid aspect. Tumoral markers were negative [CEA, CA-125, CA19-9, CA15-3, B inhibin, and alpha-fetoprotein (AFP)]. A biopsy sample revealed a dysgerminoma (**Figure 1**). She underwent radical surgery with right oophorectomy. The tumor stage was IA according to the International Federation of Gynecology and Obstetrics (FIGO) (15), so only post-surgery follow-up was indicated. At the age of 15, 2 years after the diagnosis of right ovarian dysgerminoma, a follow-up US found an inhomogeneous mass of 6 cm in the left ovary, and an MRI showed the presence of multiple nodulations of 55 mm × 45 mm and peritoneal extension of the disease. She underwent a laparoscopy surgery of left ovarian biopsy and excision of peritoneal lesions and a rectal lesion. Histological examination revealed a dysgerminoma with peritoneal and lymphonodal involvement (IIIC FIGO stage). The patient was started on chemotherapy with PEB (Etoposide, Cysplatin, and 1 Bleomycin) (16). The computed tomography (CT) scan after three courses of PEB showed a reduction in left ovary volume with a reduction in solid component of the lesions, an increase in cystic parts, and no more present peritoneal lesions nor peritoneal effusion. The patient is now well, and the abdominal residue is under strict clinical and radiological surveillance.

## Case 2

We report the case of a 19-year-old female adolescent from Iran, born full-term to non-consanguineous parents without a family history of IEI. Her paternal grandfather had a history of gastric adenocarcinoma. Since the age of 6 months she had recurrent otitis media and upper respiratory tract infections. She later developed chronic enteropathy, pustular eczema, pan-sinusitis, chronic cough, and generalized lymphadenopathy until she was referred for immunological investigation and diagnosed with common variable immunodeficiency (CVID) at age 13 years when she presented with lymphoid interstitial pneumonia and bronchiectasis [IgG, 1.3 g/L; IgA, <0.1 g/L; IgM, <0.22 g/L; white blood cells (WBC), 9,200/µL; lymphocytes, 2,690/µL; CD3+ T cells, 58.4%; CD4+ helper T cells, 16.6%; CD8+ cytotoxic T cells, 30.8%; and CD19+ B cells, 4%), and intravenous Ig (IVIg) replacement was commenced. At age 17 years, she was referred to a gynecologist due to secondary amenorrhea, and a transvaginal US at that time revealed a well-defined cyst arising from the left ovary. Pelvic lymph nodes and the tumor were removed, and an omentectomy was done. Postoperative pathology revealed poorly differentiated serous cystadenoma without infiltration to the capsule. Chemotherapy with paclitaxel and cisplatin was advised for the remnant disease after surgery without any side effects or signs of viral reactivation. Genetic evaluation of the patient was performed using the standard whole exome sequencing, and among the known IEI genes, the patient only carried one heterozygous pathogenic splicing mutation in the *PIK3R1* gene (c.1425 + 1 G>A), skipping exon 11 of the protein, the most frequent mutation (14), identified before in some patients with APDS type 2. Unfortunately, the patient was diagnosed with diffuse large B-cell lymphoma (DLBCL) 10 months after the last chemotherapy with multiple head and neck lymphadenopathy and hepatosplenomegaly, which led to obstruction of the upper airway and thoracostomy. Despite the initiation of chemotherapy according to the R-CHOP protocol (rituximab, cyclophosphamide, doxorubicin, vincristine, and prednisone), the patient responded poorly to treatment, and she died due to respiratory failure.

## Case 3

The third patient is a 12-year-old girl from the Netherlands, born full-term parents after an uneventful pregnancy to healthy non-consanguineous parents without a family history of IEI. She was diagnosed as APDS1 with the same *de novo* heterozygous mutation in *PIK3CD* observed in Case 1 (c.3061G>A). She presented some of the typical clinical features of APDS: recurrent episodes of benign lymphadenopathy, dermatological manifestation (eczema), recurrent episodes of otitis media, and mild bronchiectasis on CT scan. The immunological assessment showed normal IgG, also before subcutaneous immunoglobulin supplementation (SCIG), slightly low IgA and high IgM (ranging from 2.2 to 2.5 g/L), lymphopenia, and a typical pattern of high transitional B cells with low T naive and central memory subsets. The treatment strategy for the IEI in this patient was first prophylaxis with cotrimoxazole and subcutaneous Ig (SCIg) supplementation; some months later, she started Sirolimus, and recently, she started a specific PI3Kδ inhibitor (Leniolisib) treatment. At the age of 7, she underwent an ileocecal resection because of intussusception with a lymphonodal hyperplasia. At the age of 8, a dysgerminoma of the right ovary was found by routine ultrasound of the abdomen. The clinical and para-clinical exams performed for the staging showed that the contralateral (left) ovary was involved, and possible malignant cells were found in ascites and she was staged FIGO stage IC3. The patient received chemotherapy according to MAKEI protocol (17) with two courses of cisplatin and etoposide. Three years later, a torsion of the left ovary revealed the recurrence of dysgerminoma. Neoadjuvant chemotherapy was initiated with three courses of Carboplatin, Etoposide, and Bleomycin (18). The ovariectomy after chemotherapy showed necrosis on pathological examination. She is now almost 3 years after diagnosis, and clinical and radiological follow-up shows no signs of recurrence.

## Case 4

This sporadic index case born from non-consanguineous parents of Caucasian descent presented with upper and lower airways infections at 3 months old and benign diffuse lymphadenopathy and hepatosplenomegaly. Initially, immunological workup showed polyclonal hypergammaglobulinemia with high plasma levels of IgG and mild elevation of plasma IgM. She was diagnosed with IEI at the age of 5 years as her respiratory evaluations showed bronchiectasis, and her immunological workup showed IgG of 3.64 g/L, IgA of 0.55 g/L, and IgM of 3.40 g/L, partly due to an exudative enteropathy caused by a chronic inflammatory colitis. In addition to antibiotic prophylaxis and physiotherapy, immunoglobulin replacement was initiated with increasing dosages up to 2 g/kg of body weight/month because of high enteric protein loss. She subsequently developed an immune thrombocytopenic purpura (ITP), and she presented an intussusception at age 9 years old because of her benign diffuse lymphadenopathy and chronic EBV replication. She was then treated with azathioprine and rituximab. At age 15, she developed a chronic hepatopathy and a transient extra-membranous glomerulonephritis that resolved spontaneously. She subsequently was diagnosed with APDS1 (PIK3CD, c.3061G>A). At the age of 18 years, she presented with abdominal pain, and a US showed a right ovary lesion. Thus, an MRI was performed for better description of the lesion, and a biopsy sample revealed a unilateral ovarian dysgerminoma. She first underwent surgery (right ovariectomy and omentectomy in 2010). She relapsed a few months later, and she was treated with chemotherapy (etoposide and cisplatin); she experienced several septic shocks leading to intensive care unit admission. As she was diagnosed with a ganglionar relapse 3 months later, she underwent radiotherapy, which was successful. At age 29, she was diagnosed with DLBCL and treated with R-mini-CHOP (rituximab, reduced dose of cyclophosphamide, doxorubicin, vincristine, and prednisone). She relapsed 3 years later, and died at age 32 despite onco-hematological treatment.

## Case 5

This index case was born from non-consanguineous parents of Northern African descent. A paternal aunt experienced a lymphoma. Her three siblings have an uneventful medical history. In the first year of life, she experienced recurrent upper and lower airway infections that led to bronchiectasis. She was diagnosed with IEI at age 6 years with features of non-autoimmune mild thrombocytopenia and diffuse lymphadenopathy with splenomegaly. Immunological workup showed mild hypo IgG of 6.4 g/L, no IgA, and mild elevated IgM (2.7 g/L), with low IgG2 plasma levels (0.12 g/L). There were no detectable tetanus, diphtheria, and pneumococcus antibodies. She also had a mild lymphopenia (1,200/µL) with low naive CD4, B, and NK cells. Immunoglobulin replacement was started. She subsequently was diagnosed with APDS1 (PIK3CD, c.3061G>A) at age 12. At age 13, she was diagnosed with ovarian dysgerminoma. Her FIGO stage was 1A. Pathology showed a voluminous ovary due to edematous and angiomatous fibrosis with some CD3+T cells and CD68+ macrophages and histiocytes scattered in the tissue. Neoplastic cells expressed octamer transcription factor (OCT), and CD117, CD10, AFP, HCG, PLAP, Vimentin, AEI-AE3, and CJ20 were negative. She underwent ovariectomy and (MRI) surveillance on a regular basis. The oncological follow-up was uneventful. She underwent a hematopoietic stem cell transplantation (HSCT) with a matched sibling donor at age 22. She died 8 months later of acute exacerbation of chronic obstructive pulmonary disease.

## Discussion

These five cases present with frequent and well-known clinical and immunological features of APDS (5, 6, 19), which are summarized in **Table 1**. The most common malignancies reported in APDS are B-cell lymphoma (diffuse large B-cell lymphoma), Hodgkin lymphoma, and marginal zone B-cell lymphoma. Non-hematological malignancies have also been reported. In the Update of ESID Registry published in 2023 (20), seven non-lymphoid tumors were reported out of 170 patients: two ovarian neoplasms not otherwise specified, one papillary renal cell carcinoma, one malignant neoplasm of the submandibular gland, one hepatocellular carcinoma, one breast ductal carcinoma *in situ*, one papillary thyroid carcinoma, and one rhabdomyosarcoma. Ovarian neoplasms not otherwise specified represent 28.6% (2/7) of solid tumors reported. Ovarian neoplasms reported were 2/87 female patients (prevalence, 2.3% in female population); both of these patients are part of our five described cases. The number of patients with APDS in the ESID registry as of 8/01/2025 is 235 alive, 125 male and 110 female patients (168 APDS1 and 67 APDS2). Ovarian neoplasms are reported in 5/110 female patients. Underreporting of cases is possible, as documentation in the registry is not up to date for all patients. Furthermore, some female patients are too young to develop an ovarian malignancy; the median age in this female population is 21 years with a minimum of 3 years and a maximum of 59 years. To our knowledge, at the moment, three additional cases of ovarian dysgerminoma in female patients with APDS are reported in the literature. The first one was from the United States Immunodeficiency Network (USIDNET) in 2016; she was a female patient with APDS1 who was reported to have an ovarian dysgerminoma (11). The second one was a 37-year-old woman with APDS1 syndrome described in 2017 by Wentink et al. (12), and the third one was a 9-year-old girl with APDS1 from a Chinese cohort of 40 patients with APDS (13) both with a diagnosis of dysgerminoma. It is interesting that all three ovarian tumors reported in the literature and four of five of our patients had a dysgerminoma; all patients with dysgerminoma also had APDS1 while our only patient with a serous cystadenoma had APDS2. From these preliminary data, it would therefore appear that the most frequent ovarian tumor in patients with APDS (seven out of eight patients) is dysgerminoma. In the general population, ovarian neoplasms have a reported annual incidence of 2.6 cases per 100,000 children and adolescents (21). Germ cell tumors are the most common ovarian neoplasms in the pediatric population, accounting for approximately 60%–80% of cases. Dysgerminoma, a malignant germ cell tumor, is the most common ovarian malignant neoplasm in childhood and adolescence (22). The majority of cases of dysgerminoma occur in the second and third decades of life, although 10% of cases are diagnosed during the first decade (23). Our cases 1, 4, and 5 developed dysgerminoma in the second decade, while case number 3 developed dysgerminoma at the age of 8, an age less common for presentation. With our data, we cannot say if dysgerminoma in APDS can be considered to develop earlier than in the general population. In the general population, dysgerminomas are bilateral in 10%–15% of cases (24); in our series two of the five patients developed a bilateral dysgerminoma. Epithelial ovarian tumors are rare in children and account for only 15%–20% of all pediatric ovarian neoplasms, and this type of tumor is more typical of the adult population (25). Our patient (case 2) developed a serous cystadenoma at the age of 17, earlier than in the general population. Some types of pediatric ovarian neoplasms have been associated with specific cancer predisposition syndromes, including Peutz–Jeghers syndrome, hereditary leiomyoma renal cell carcinoma syndrome, DICER1, rhabdoid tumor predisposition syndromes, nevoid basal cell carcinoma syndrome, and von Hippel–Lindau syndrome (26, 27). It is known that PI3K/AKT signaling is involved in important physiological and pathophysiological functions that drive tumor progression such as metabolism, cell growth, proliferation, angiogenesis, and metastasis (28, 29). There is currently no specific evidence providing a causal link between PI3Kδ and ovarian tumors. However, mutations in the PI3K–AKT–mTOR pathway are frequently observed in ovarian cancer, with evidence that this signaling pathway plays a prominent role in ovarian tumorigenesis and chemo- and radiotherapy resistance (30). Epithelial ovarian cancer exhibits a strong association with defects in PIK3CA and PTEN; it has been reported that the genomic alterations in PIK3CA are found in >20% of all ovarian cancers (31–33). In particular, in ovarian cancer it is described the mutation H1047R in PIK3CA that encodes for p110alpha subunit of PI3K (34). All the APDS1 patients with dysgerminoma in this series and literature (seven cases) had the same mutation on PIK3CD: c.3061G>A (E1021K), the most frequent mutation described in APDS1 (14). The E1021K mutation in PIK3CD and H1047R mutation in PIK3CA are similar in functional localization and biological effects: E1021K is located in the C loop of the kinase domain resulting in constitutive activation, and the H1047R mutation similarly affects the activation loop of kinase domain leading to hyperactivation (2, 35, 36). This aspect could be relevant to understand the biochemical mechanism of ovarian malignancy susceptibility in APDS1. Somatic mutations in the PIK3R1 gene, the gene involved in APDS2, were found in patients with carcinoma located in the ovary, large intestine, stomach, and malignant melanoma (5). In a study published in 2018, they detected recurrent focal deletions and enrichment in the PI3K/AKT pathway in germinal tumors (yolk sac tumors) (37). Thus, a role for germline mutations in the pathway in determining a predisposition to ovarian neoplasms can be speculated. Furthermore an ovarian neoplasm detected during childhood may be the first manifestation of APDS: in case number 2, the patient received the diagnosis after the development of the ovarian neoplasm. In this case, she had yet some signs and symptoms of IEI but in the case of nuanced signs of IEI, the initial presentation with cancer is not excluded. Our patients who underwent chemotherapy had good tolerance except patient 4. This patient had serious infectious complications probably due to her comorbidities given that she did not present substantial immunological differences compared to the other patients. Two of our patients (cases 2 and 4) developed a second malignancy, both a diffuse large B-cell lymphoma. This is to remark the important cancer predisposition in this syndrome. With this case series, we want to highlight the importance of considering the predisposition to non-hematological malignancies in APDS patients and in particular ovarian cancer in women with this disease. A limitation of our study is that the specific somatic mutation in the tumor tissue has not been investigated, but we inted to do in the future. Based on our experience, we suggest annual abdominal and pelvic ultrasound in female patients starting from childhood and, if necessary, MRI, tumor markers (CA-125, BHCG, AFP, and LDH) and a gynecological consult. Furthermore, in female APDS patients presenting with abdominal pain, an ovarian tumor has to be in the differential diagnosis, and further investigations need to be performed to rule out this diagnosis even if they already have a mesenterial lymphoproliferation that could explain the abdominal pain. In the future, it could be interesting to study if target therapy with inhibitors of the PI3K/AKT/mTOR pathways used to treat symptoms of APDS could also play a role in reducing the risk of ovarian tumorigenesis or, in association with conventional chemotherapy, in the first-line treatment of tumors in APDS patients.

**Table 1:** Main clinical and immunological features of APDS in the five cases reported and three cases of literature (cases 6-7-8. References 11–13).

	Case 1	Case 2	Case 3	Case4	Case 5	Case 6 (ref^11^)	Case 7 (ref^12^)	Case 8 (ref^13^)
Age at diagnosis of ovarian tumor (years)	13	17	8	18	13	NA	37	9
Type of ADPS (1/2)	1	2	1	1	1	1	1	1
Genetic mutation	PIK3CD c.3061G>A (p.Glu1021Lys/ E1021K)	PIK3R1 c.1425 + 1 G>A	PIK3CD c.3061G>A (p.Glu1021Lys/ E1021K)	PIK3CD c.3061G>A (p.Glu1021Lys/ E1021K)	PIK3CD c.3061G>A (p.Glu1021Lys/ E1021K)	PIK3CD c.3061G>A (p.Glu1021Lys/ E1021K)	PIK3CD c.3061G>A (p.Glu1021 Lys/ E1021K)	PIK3CD c.3061G>A (p.Glu1021Lys/ E1021K)
Clinical feature								
Infections								
Respiratory tract infections	Yes	Yes	Yes	Yes	Yes	NA	NA	Yes
Persistent/chronic viral infections	No	No	No	No	No	NA	NA	No
Ocular infections	No	No	No	No	No	NA	NA	No
Digestive infections	No	No	No	No	No	NA	NA	No
Mucocutaneus infections	No	Yes	Yes	No	No	NA	NA	No
CMV – EBV- viremia by PCR	No	No	No	Yes (EBV)	No	NA	NA	No
Benign Lymphoproliferation								
Lymphadenopathy	Yes	Yes	Yes	Yes	Yes	NA	Yes	Yes
Splenomegaly	Yes	Yes	No	Yes	Yes	NA	No	Yes
Hepatomegaly	No	Yes	No	Yes	Yes	NA	No	No
Autoimmunity						NA		
Cytopenia	No	No	No	Yes	No	NA	NA	Yes
Others	No	No	Autism, intussusception with lymphonodal hyperplasia	No	Recurrent oral aphthous	NA	NA	No
Malignancy	Bilateral ovarian dysgerminoma	Ovarian serous cystadenoma Diffuse large B cell lymphoma	Bilateral ovarian dysgerminoma	Unilateral ovarian dysgerminoma Diffuse large B cell lymphoma	Unilateral ovarian dysgerminoma	Unilateral ovarian dysgerminoma	Unilateral ovarian dysgerminoma	Unilateral ovarian dysgerminoma
Enteropathy	Yes	Yes	No	Yes	No	NA	NA	No
Dermatological manifestations	No	Yes (eczema)	Yes (eczema)	No	No	NA	NA	No
Neurodevelopmental delay	Yes	No	No	No	No	NA	NA	No
Short stature	No	No	No	No	No	NA	NA	No
Immunological features								
IgM g/L	2,05 (normal)	<0,22 (low)	2.93 (high)	2.72 (slightly high)	2.7 (slightly high)	NA	NA High	NA
IgG g/L	16,8 (hight)	1,30 (low)	7.0 (normal)	15.31 (hight)	6.4 (low)	NA	NA Low	NA
IgA g/L	1,68 (normal)	<0,1 (low)	0.48 (slightly low)	1.68 (normal)	<0,1 (low)	NA	NA Low	NA
WBC (/µL) Total lymphocytes (/µL) Blood T cell subsets T cells (/µL) T cells in total lymphocytes (%) CD4 T cells in total lymphocytes (%) CD8 T cells in total lymphocytes (%) Naïve CD4 T cells (/µL) Naïve CD4 T cells in total CD4 T cells(%) CD4+CD8+ T cells in total T cells (%) CD4−CD8− (double negative α/β T cells) in total T cells (%) Natural Killer cell subsets NK cells (/µL) Blood B cell subsets Total B cells (/µL) B cells of total lymphocytes (%) Naïve B cells of total B cells (%) Transitional B cells of total B cells (%) Memory B cells of total B cells (%) Class switched memory B cells of total B cells (%) IgM only memory B cells of total B cells (%)	8,600 2,600 2,226 85,6 31,8 47,6 NA NA 1,6 9,4 91 241 9,3 NA NA NA NA NA	9,200 2,668 1,558 58.4 16.6 30.8 92 37 NA 5.7 430 105 4 62 17 1 1 3	5,450 1,120 638 57 30 NA 90 27 NA 2.6 160 312 28 78 25 2 5 3	10,200 1,500 1,140 76 21 47 NA 13 NA NA 50 30 2 NA NA NA NA NA	NA 1,200 NA NA NA NA 51 NA NA 2.1 56 122 10 NA NA NA NA NA	NA NA NA NA NA NA NA NA NA NA NA NA NA NA NA NA NA NA	NA 2,100 NA NA NA NA NA NA NA NA 70 50 2,3 NA NA NA NA NA	NA NA NA NA NA NA NA NA NA NA NA NA NA NA NA NA NA NA
Alive	Yes	No	Yes	No	No	NA	NA	Yes

NA, not available.

## Data Availability

The data analyzed in this study is subject to the following licenses/restrictions: database of ESID APDS registry. Requests to access these datasets should be directed to maria.elena.maccari@uniklinik-freiburg.de.
